# AGING KNOWLEDGE AND SELF-REPORTED AGEISM

**DOI:** 10.1093/geroni/igz038.314

**Published:** 2019-11-08

**Authors:** Katie E Cherry, Marla J Erwin, Priscilla D Allen

**Affiliations:** Louisiana State University, Baton Rouge, Louisiana, United States

## Abstract

The term, ageism, refers to any form of personal or institutional prejudice or discrimination based on chronological age. Ageism may encompass attitudes and prejudices, as well as behaviors, highlighting the complex nature of ageist behaviors observed among students and professionals alike (Allen, Cherry, & Palmore, 2009). We examined the prevalence of self-reported ageist behaviors in a sample of college students who ranged in age from 18 to 44 years to test the hypothesis that aging knowledge would be associated with self-reported ageist behaviors (positive and negative). The study sample was comprised of adults who were enrolled in classes at Louisiana State University (n = 110). Most of these students were traditional aged college students (18-25 years old). Participants completed the Relating to Older People Evaluation (ROPE; Cherry & Palmore, 2008), the Facts on Aging Quiz (FAQ; Palmore, 1998), and the Knowledge of Memory Aging Questionnaire (KMAQ: Cherry et al., 2003). Results indicated that positive ageist behaviors were more frequent than negative ageist behaviors. Men endorsed positive and negative ageism items more than women reported. Follow-up analyses on participants’ responses to the two aging knowledge questionnaires showed that increased knowledge of aging was significantly correlated with diminished reports of negative ageist behaviors, after controlling for age and gender. These results imply that self-reported ageist behaviors are associated with aging knowledge. Strengthening college curricula by including course offerings in adult development and aging may improve self-reported ageist behaviors among college students.

